# Skin Immunosenescence and Type 2 Inflammation: A Mini-Review With an Inflammaging Perspective

**DOI:** 10.3389/fcell.2022.835675

**Published:** 2022-02-24

**Authors:** Bangtao Chen, Jing Yang, Yao Song, Daojun Zhang, Fei Hao

**Affiliations:** ^1^ Department of Dermatology, Chongqing University Three Gorges Hospital, School of Medicine, Chongqing University, Chongqing, China; ^2^ Department of Dermatology, The Third Affiliated Hospital of Chongqing Medical University, Chongqing, China

**Keywords:** immunosenescence, inflammaging, skin aging, dermatosis, mini-review and challenges

## Abstract

Skin-resident stromal cells, including keratinocytes, fibroblasts, adipocytes, and immune cells including Langerhans cells, dendritic cells, T cells, and innate lymphoid cells, and their functional products work in concert to ensure the realization of skin barrier immunity. However, aging-induced immunosenescence predisposes the elderly to pruritic dermatoses, including type 2 inflammation-mediated. Inflammaging, characterized by chronic low level of pro-inflammatory cytokines released from senescent cells with the senescence-associated secretory phenotype (SASP), may drive immunosenescence and tangle with type 2 inflammatory dermatoses. The present mini-review summarizes current evidence on immunosenescence and type 2 inflammation in the skin and further focuses on future needs from an inflammaging perspective to clarify their complexity.

## Introduction

**FIGURE 1:**
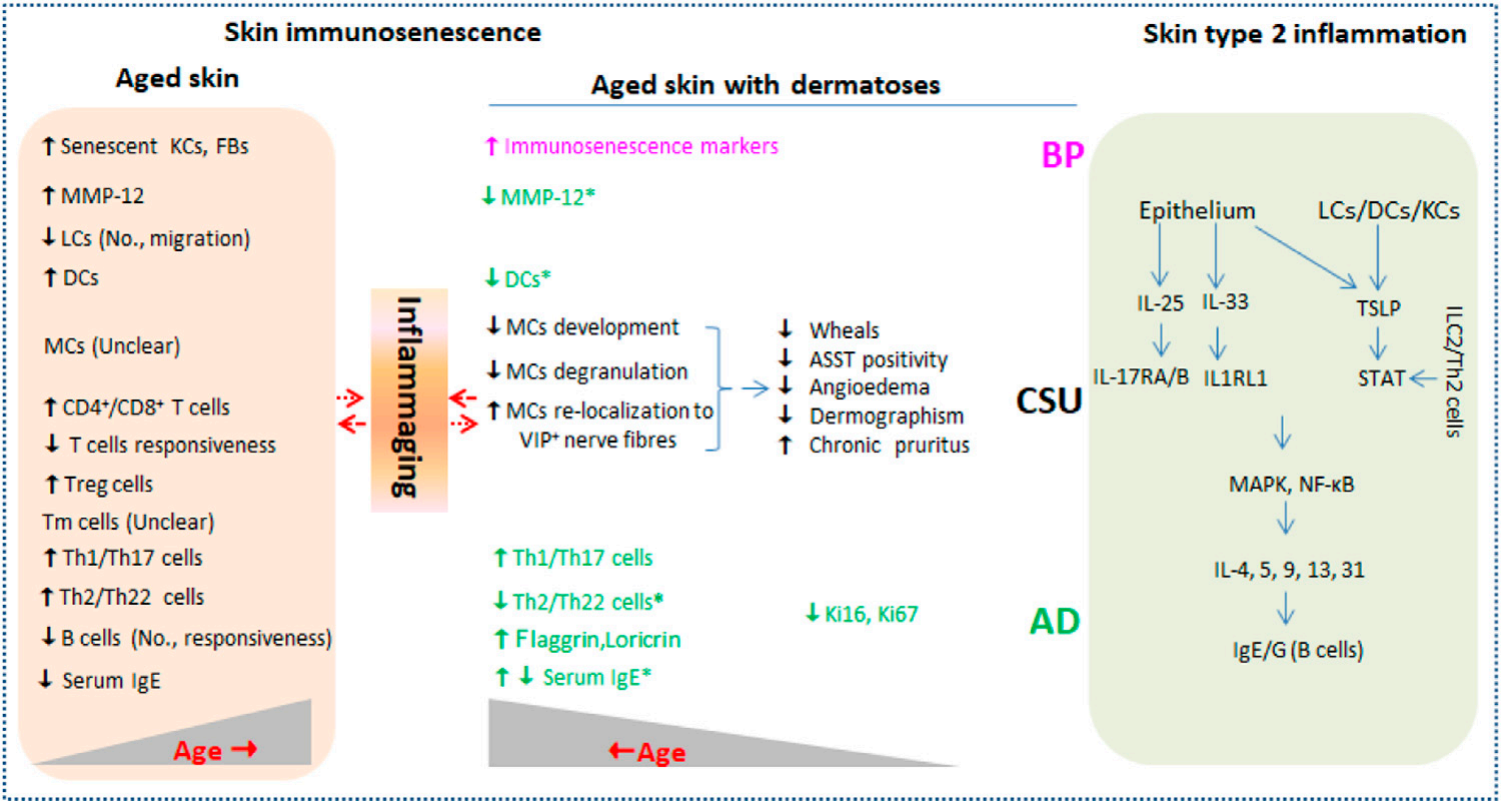
Current evidence of immunosenescence in aged skin with or without type 2 inflammation dermatoses. Type 2 inflammation dermatoses such as AD, CSU, and BP are driven by key cytokines including IL-25, IL-33, and TSLP released from damaged epithelium and LCs/DCs (right column). Skin barrier inevitably undergoes characteristically immunological changes (skin immunosenescence) during aging in healthy individuals (left column) and in individuals affected by type 2 inflammation dermatoses. Inflammaging that is characterized by low level of pro-inflammatory cytokines including CRP, IL-1β, IL-6, and TNF-α produced by senescent skin cells may be in complex interaction with the two conditions. No., number; KCs, keratinocytes; FBs, fibroblasts; LCs, Langerhans cells; DCs, dendritic cells; MCs, mast cells; ILC2, innate lymphoid cell 2; Tm, memory T cells; Ig, immune globulin; MMP-12, matrix metalloproteinase 12; ASST, autologous serum skin test; TSLP, thymic stromal lymphopoietin; STAT, signal transducer and activator of transcription; IL1RL1, IL-1 receptor-like 1; AD, atopic dermatitis; CSU, chronic spontaneous urticaria; BP, bullous pemphigoid.

The skin is the largest active immune organ, covering the body’s outermost layer and performing the function of resisting external stimulus, thus maintaining skin homeostasis. Skin barrier inevitably undergoes characteristically immunological declines with advancing age, termed skin immunosenescence. Higher incidences of many dermatoses such as infectious diseases, non-communicable autoimmune diseases, and cutaneous malignancies, and more pathological states such as unspecific itchiness and delayed wound healing are observed in the elderly alongside immunosenescence (Farage et al., 2009). Senescent cells remain senescence-associated secretory phenotype (SASP) secreting low-level pro-inflammatory cytokines including CRP, IL-1β, IL-6, and TNF-α, which is usually referred to inflammaging (Lopes-Paciencia et al., 2019; Fitsiou, et al., 2021). Type 2 inflammatory dermatosis such as atopic dermatitis (AD), chronic spontaneous urticaria (CSU), and bullous pemphigoid (BP) frequently affect the elderly and are presumed to be correlated with skin immunosenescence. Moreover, the diseases affecting the elderly are prone to more severity, therapeutic resistance, and longer duration. The current mini-review focuses on skin immunosenescence and type 2 inflammation and present future needs from an inflammaging perspective, promising better management of type 2 inflammatory dermatosis in the elderly (Figure 1).

## Skin Barrier Immunity

Skin-resident stromal cells, including keratinocytes, fibroblasts, and adipocytes, and immune cells including Langerhans cells (LCs), dendritic cells (DCs), T cells, and innate lymphoid cells (ILCs) together with their functional products ensure the realization of skin barrier immunity. The cells mentioned above work synergistically or antagonistically upon harmful environmental exposures challenge, leading to reinforced or compromised networks protecting the skin against damage or causing dermatosis, respectively.

### Stromal Cells

Epidermal keratinocytes express and secrete antimicrobial ribonuclease RNase 7 and antimicrobial peptides adrenomedullin, β-defensins, and cathelicidin upon recognizing pathogenic components *via* its constitutive expressions of toll-like receptors (TLRs) on the cellular surface (Miller and Modlin, 2007; Köllisch, et al., 2005). In addition, keratinocytes function in the presentation of antigen from CD4^+^ to CD8^+^ T cells and promote tissue repair *via* chemokine (IL-1β, IL-8, and CCL20)-mediated leukocyte recruitment during early wound healing (Black, et al., 2007; Li, et al., 2017; Sperling, et al., 2012). They also serve as key sites for UVB-catalyzed production of active vitamin D3 (Zbytek, et al., 2008). Dermal fibroblasts are not only key to supporting wound healing through the secretion and remodeling of extracellular matrix (ECM) but also essential for facilitating innate immune response to microbial infections by secreting cytokines and chemokines with involvement of toll-like receptor activation (Ghetti, et al., 2018; Cole, et al., 2018; Haniffa, et al., 2007; Bautista-Hernández, et al., 2017). Interestingly, fibroblasts were uncovered to inhibit T-cell proliferation and induce the production of immunoregulatory cytokines such as IL-10 (Haniffa, et al., 2007). Moreover, adipocytes differentiated from dermal fibroblasts upon *Staphylococcus aureus* infection can also produce the antimicrobial peptide cathelicidin (Zhang, et al., 2015).

### Immune Cells

LCs, the mononuclear phagocyte within the epidermis, not only produce antimicrobial peptide hBD3 and initiate a local immune response mainly by presenting antigens to T cells (Ferris, et al., 2013; Atmatzidis, et al., 2017; Pilkington, et al., 2018a), but also migrate to skin regional lymph nodes for enhancing immune response to exogenous antigens and promoting tolerance to self-antigens (West and Bennett, 2018; Atmatzidis, et al., 2017).

DCs and macrophages, the mononuclear phagocyte located in the dermis, are also the sentinels of the innate immunity working similarly to epidermal LCs. Dermal DCs comprise CD1c^+^ or CD141^+^ myeloid and plasmacytoid forms, while the latter is hardly observed in steady-state skin (Collin and Bigley, 2018). Compared with their blood counterparts, normal dermal DCs associated with T-cell proliferation displayed an activated phenotype with increased expression of co-stimulatory receptors (McLellan, et al., 1998). Dermal macrophages are specifically labeled with CD163, and the cells also contribute to wound and nerve healing by suppressing inflammation upon tissue injury (Kolter, et al., 2019).

The same as skin LC and DC, B cells found in healthy skin are integral for presenting antigen at low concentration to T cells (Geherin, et al., 2012). Moreover, skin B cells also modulate inflammation response by secreting pro- or anti-inflammatory mediators (Debes, and McGettigan, 2019).

Skin-resident T cells derived from T cells differentiated and matured in the thymus *via* migration through the lymphatic or circulatory system. Phenotypically, 80%–90% of the skin T-cell pool is memory T (Tm) cells, and the remaining is recirculating T cells (Nguyen, et al., 2019). Tm cells have stronger immune surveillance against reinvasions, and expressions of CD69 and CD103 on cell surface commonly characterize this type of T cells (Mackay, et al., 2013). The number of CD4^+^ Tm cells is three and six times that of CD8^+^ Tm cells in the epidermis and dermis, respectively (Watanabe, et al., 2015). With a memory skin-resident phenotype inducing immune tolerance, Foxp3^+^ regulatory T cells (Tregs) are in close proximity to hair follicles where skin commensal-metabolized short-chain fatty acid sodium butyrate or UVB light increases Foxp3^+^ expression in non-Tregs or drive Foxp3^+^ Tregs proliferation (Schwarz, et al., 2017; Yamazaki, et al., 2014; Scharschmidt, et al., 2017). Overall, αβ T cells dominate in the skin as in circulation (Nielsen, et al., 2017).

Cutaneous ILCs located in the epidermis and dermis are newly identified immune cells whose function is not fully understood, but influxes of ILC2 in AD and LC1/3 in psoriatic plaques were demonstrated (Brüggen, et al., 2016; Akdis, et al., 2020). Neutrophils are seldom in the skin, while they can infiltrate the skin upon exposure to a harmful stimulus (Rijken, et al., 2005). In addition, allergens or inflammatory irritants can induce the release of histamine and inflammatory mediators from cutaneous mast cells (MCs), mediating wheals and itch onset (Otsuka, and Kabashima, 2015).

## Skin Immunosenescence

The skin goes roughly through stages of immaturity, maturation, and decline over lifespan as with all other organs. Although incredibly durable, aging still causes skin structure and function changes, termed skin aging. This process is usually exaggerated by extrinsic exposures such as UVR. Morphologic and related functional changes in chronologically or intrinsically aged skin were summarized in a review conducted by Zouboulis, and Makrantonaki, (2011). In particular, immunosenescence contributes to the increased susceptibility to skin disorders with malignancies, infections, and autoimmunity in the elderly. Skin immunosenescence refers to declines in function or number of all skin cells responsible for immune surveillance (Corsini, et al., 2009). Senescent cells, promoted by telomere shortening and genome instability, remain SASP secreting low level of pro-inflammatory cytokines including IL-1β, IL-6, and CRP, thus altering the skin’s microenvironment (Lopes-Paciencia, et al., 2019; Fitsiou, et al., 2021). Presumably, skin inflammaging characterized by chronic low-level inflammation is believed to be the main driver for remodeling the immunological response in senescent skin cells (Ghosh, and Capell, 2016).

### Stromal Cell Senescence

Dermal senescent fibroblasts accumulated with age and displayed SASP rich, thus maintaining inflammaging phenotype (Wlaschek, et al., 2021). Such changes contribute to disruptions of collagen homeostasis, delayed wound healing, and increased likelihood of skin tumorigenesis; however, its antibacterial immunity loss caused by aging has been associated with impaired adipocyte differentiation (Zhang, et al., 2019; Wasko, and Horsley, 2019). Compared with fibroblasts, the impact of accumulated senescent keratinocytes in the epidermis on inflammaging or antibacterial immunity is limited due to its higher turnover rate (Pilkington, et al., 2021).

### Immune Cell Senescence

In aged skin, decreased proliferation of *in situ* LC progenitors causes a reduced number of LCs, and LCs are also less able to migrate from the epidermis in response to harmful stimulus due to the declined availability of local IL-1β, which collectively contributes to impaired skin barrier integrity and diminished antimicrobial and tumor cell defense (Pilkington, et al., 2018b). In addition, LC-mediated skin barrier perturbation may facilitate the onset of skin inflammaging by initiating cytokine release from cutaneous cells (Wittmann, et al., 2014).

To some extent, the state of thymus and T cells in circulation are implicated in many dermatoses, which also reflect the profiles of skin-resident T cells. In geriatric individuals, circulating T cells in total number remain unchanged, accompanied by reduction of naive T cells due to thymic involution and increase of Tm cells since the prolonged exposure to external substances over the lifespan (Thomas, et al., 2020). However, little is known regarding the changes in skin-resident Tm cells during aging. A higher ratio of CD4^+^ to CD8^+^ T cells was found in aged skin than in young skin, indicating a more severe pro-inflammatory response phenotype (Zuelgaray, et al., 2019). Cytokines during inflammaging can be Th2 pattern dominant with an increased incidence of allergic diseases and Th1 pattern dominant with a higher frequency of chronic infections and neoplastic diseases. It was reported that Tregs numbers and immunosuppressive receptor PD-1 increased in aged skin, thus causing reactivation of infectious diseases or skewing inflammatory microenvironment by suppressing both Th1 and Th2 responses (Lages, et al., 2008). Additionally, a diminished response of T cells to specific antigens in advanced age may collectively explain why the chronic low-level inflammation characterizes the state of inflammaging (Bektas, et al., 2017).

Aging-related changes in skin B cells are similar to skin T cells except that B cells from the elderly are less efficiently stimulated. Thus, antibody generation decreases, and immune response to vaccines and antigens is weakened (Pinti, et al., 2016).

## Immunosenescence and Type 2 Inflammatory Dermatosis

Type 2 inflammation phenotypes in skin and circulation are usually in traffic with each other and remain consistent. IL-25, IL-33, and thymic stromal lymphopoietin (TSLP) released from damaged epithelium directly activate the production of IL-4, 5, 9, 13, and 31 from ILC2 and Th2 cells, thus characterizing type 2 inflammation immunity (Akdis, et al., 2020). Both IL-25 and IL-33 can activate MAPK and NK-κB signaling pathways *via* binding to IL-17RA/B and IL-1 receptor-like 1 (IL1RL1), respectively (Akdis, et al., 2020; Deng, et al., 2021). By activating signal transducer and activator of transcription (STAT), skin LC/DC-derived and keratinocyte-derived TSLP are critical for Th2-type immune responses and mediating pruritus exacerbation, respectively (Kim et al., 2013). In addition, humoral immunity characterized by allergen-specific antibody IgE or autoantigen-specific autoantibody IgG matured by IL-13 and IL-4 is also involved in type 2 inflammatory dermatosis such as AD, CSU, and BP (Gandhi, et al., 2016).

### Atopic Dermatitis

Globally, 10% of adults and 1%–3% of elderly populations are troubled by AD (Lloyd-Lavery, et al., 2019; Williamson, et al., 2020). Moreover, the increasing predisposition of late AD development in older adults is due to exposure-induced epidermal barrier malfunction and immunosenescence-caused chronic itch in advanced age. The core of AD is skin inflammation involving IgE produced by B cells and inflammatory mediators of T-cell origin, while Th2 cytokines dominate in the inflammation milieu (Tanei, and Hasegawa, 2016; Tanei, et al., 2013). Th2/Th22 cytokines in skin increase during aging in healthy individuals, while the opposite phenomenon is observed in older AD patients (Bocheva, et al., 2021; Gittler, et al., 2012). With age progressing, Th1- and Th17-related mediators in lesioned and non-lesioned skin in individuals suffering from AD are markedly increased, as observed in healthy adults (Bocheva, et al., 2021; Gittler, et al., 2012). Zhou et al. showed that inflammatory DCs in the skin and cutaneous expression of matrix metalloproteinase 12 (MMP-12) were reduced in both affected and unaffected skin in AD with aging (Agrawal, et al., 2012; Zhou, et al., 2019). Reduction in specific and total serum IgE with aging in patients with allergic rhinitis, asthma, or insect allergy implies a decreasing proportion of extrinsic atopy among older adults; however, the association between serum IgE and aging in AD patients remains inconsistent (Zhou, et al., 2019; Mediaty, and Neuber, 2005). In addition, aging-related increment in terminal keratinocyte differentiation markers (filaggrin and loricrin) and decrement in epidermal hyperplasia markers (Ki16 and Ki67) were also observed in AD (Zhou, et al., 2019), which might be attributable to attenuation in the Th2/Th22 cytokine axes (Boniface, et al., 2005); moreover, it reveals a critical role for crosstalk between immune cell senescence and stromal cell-mediated immunity impairment in severity of geriatric AD.

### Chronic Spontaneous Urticaria

Traditionally, CSU is an allergic dermatosis mediated by degranulation and histamine released from skin MCs or basophils (Bracken, et al., 2019). As mounting CSU patients show antihistamine resistance, it is supposed to be T cell-mediated with emerging evidence that concentrations of circulating cytokines released from Th1/Th2 and Th17 cells correlated positively with disease severity in our previous study (Chen, et al., 2018). Kay et al. added the finding that increased expressions of IL-25, IL-33, and TSLP in skin wheals of patients with CSU further accurately characterize the pathogenesis and categorize it as type 2 inflammatory dermatosis (Kay, et al., 2015; Vadasz, and Toubi, 2015). In retrospective investigations performed in localized areas, older CSU patients made up 9.4%–25% of the CSU population. Furthermore, fewer wheals, lower rates of ASST positivity, angioedema, and dermographism, and more comorbidities were reported in elderly patients with CSU diagnosis (Chen, et al., 2012; Magen, et al., 2013). The atypical symptoms are pertinent to aging-related immunosenescence. For one thing, stromal-cell functional impairment with aging was proved to cause a decline in MC development (Tsuboi et al., 2012). For another, skin MCs accumulated while their degranulation capability was reduced with aging. Furthermore, they re-localize to the papillary dermis, where MCs keep in closer proximity to macrophages and VIP^+^ nerve fibers while the association with dermal vasculature is weakened (Pilkington, et al., 2019). Unfortunately, little is known regarding alterations of number, function, and crosstalk among MCs, basophils, and T and B cells in elderly individuals with CSU.

### Bullous Pemphigoid

BP, an autoimmune blistering dermatosis in the elderly mediated by IgG autoantibodies to skin hemidesmosome proteins (BP180 and/or BP230) and activation of complement component C3, is characterized by urticarial plaques, tense blisters, and intractable pruritus (Bağcı, et al., 2017). In BP development, autoreactive T cells work cooperatively. Increased circulating Th2 cells and IL-4 promote B-cell proliferation, antibody production, and immunoglobulin class-switching, while skin-resident Th17 cells and IL-17 activate local neutrophil-mediated inflammatory response, thus causing tissue damage (Fang, et al., 2020; Boehncke, and Brembilla, 2019). In recent studies, specific anti-BP180/230 IgE in BP were detected by immunoassays; furthermore, positive associations between IgE content and Th2 cell-specific cytokines IL-4/-13 and symptomatic disease phenotypes were shown (Cozzani, et al., 2018; Messingham, et al., 2019). The finding that IgE-driven BP promises the therapeutic regimes using Th2 inhibitors in BP-affected frail patients with good safety and ideal effectiveness. It was reported that disease clearance or satisfactory response was achieved in 12 of 13 BP patients (an average age of 76.8 years) treated with Dupilumab, an IL-4 receptor alpha antagonist with the property of inhibiting IL-4/-13 signaling and IgE secretion (Abdat, et al., 2020). Immunosenescence-related aging is conceivably responsible for the increased incidence of BP in the elderly (Pietkiewicz, et al., 2016; Yaar, and Gilchrest, 1987). However, fewer studies focus on the effect of immunosenescence or inflammaging on pathophysiological characteristics of BP, and only a meeting paper uncovered increased markers of immunosenescence in BP patients (Noe, et al., 2015).

## Future Needs

Too many questions regarding skin immunosenescence and type 2 inflammations need to be answered.

Firstly, existing studies fail to provide direct and strong evidence for the involvement of immunosenescence in type 2 inflammatory dermatosis. Distinctive clinical features and incidences of type 2 inflammatory dermatosis between the young and the elderly are observed, and the difference is often thought to be caused by aging-related changes including immunosenescence, but direct evidence remains insufficient. As evidenced by the recent discovery of TH2-interacting fascial fibroblasts (TIFFs) in mouse and human skin, skin-resident or -infiltrating immune cells and stromal cells are complexly interacting and influence each other throughout life (Boothby, et al., 2021). They undergo structural and functional alterations simultaneously, but overall, inflammaging phenotype characterizes the skin microenvironment during normal aging. In aged individuals with type 2 inflammatory dermatosis, the relationship of the skin microenvironment with inflammaging and changes to cutaneous immunity is more complex due to repeated scratching caused by uncontrolled itchiness. Therefore, it is far-fetched to conclude that a specific pathophysiological change is independently caused by a specific senescent cell alone or the disease itself. In particular, further exploration of the associations between remolding of senescent fibroblast-released ECM and type 2 inflammation in aged skin will provide new insights into strategies used in related dermatosis.

Secondly, could skin immunosenescence contribute to systemic immunosenescence or *vice versa* from the perspective of inflammaging? A shining shared feature of type 2 inflammatory dermatoses in the young or elderly is the presence of Th2 cytokines in circulation and the lesions, and inflammation state in lesions is proposed to be orchestrated by systemic inflammation phenotype (Rafei-Shamsabadi, et al., 2019; Pezzolo and Naldi, 2020). Given this, the reverse argument is worth further considering, especially in the elderly with impaired skin barrier as epidermal abnormality in AD has been proposed to drive systemic inflammation (Elias and Steinhoff, 2008). Hu et al. showed that tape stripping-induced epidermal dysfunction led to an age-associated increase in levels of circulating inflammatory cytokines in mice (Hu, et al., 2017), and Ye et al. also provided the evidence that correction of epidermal function by emollient lowered systemic inflammaging measured by circulating levels of IL-6 and TNF-α in chronically aged human (Ye, et al., 2019). These studies may collectively support the thesis that epidermal dysfunction-mediated immunosenescence could contribute to the onset or severity of type 2 inflammatory dermatosis with systemic inflammation involved in the elderly. However, more investigations are warranted to confirm the thesis by untangling their cause and effect.

On the contrary, the complexity of systemic and tissue inflammaging can also be witnessed in the efficacy of anti-inflammaging agents. Anti-inflammaging drugs indeed hold promise for increasing healthy aging, and much effort aimed at slowing aging by targeting inflammaging has been conducted (Partridge, et al., 2020; Suggs, et al., 2014). Rapamycin, metformin, and various botanicals showed delaying the aging process by inhibiting cellular senescence dependent or independent of their anti-inflammaging properties (Partridge, et al., 2020; Suggs, et al., 2014). For example, topical rapamycin, an FDA-approved agent, showed no beneficial effects in inflammaging (Correia-Melo, et al., 2019) but improved histological appearance of aged skin by reducing fibroblast senescence and increasing collagen VII (Chung, et al., 2019; Qin, et al., 2018). As such, the correction of the impaired skin barrier by anti-inflammaging agents preventing or mitigating systemic inflammaging is meaningful and easily articulable.

Thirdly, other intrinsic drivers of skin immunosenescence or inflammaging should also be identified in terms of the organism as a whole. Changes to gut and skin microbiota, mitochondrial damage-associated molecular patterns (DAMPs), abnormal activity of coagulation and fibrinolysis, complements, and vitamin D3 deficiency during aging have also been linked with type 2 inflammation dermatoses (Nakahara, et al., 2021; Sánchez-Borges et al., 2018; Hashimoto, et al., 2020). Whether the correction of abnormalities benefits improvement of related dermatoses through immunosenescence retardation remains to be further investigated.

Lastly, skin immunosenescence can be partly determined with flow cytometry and immunohistochemistry by frequency assessments of senescent immune cells due to their end-stage differentiated and cell-specific markers; however, no techniques are available for assessing inflammaging caused by indicated senescent cells *in vivo*. It appears that all immune- or non-immune-senescent cells possess SASP properties releasing low levels of IL-1β, IL-6, TNF-α, and CRP. These pro-inflammation mediators that can shuttle through skin and circulation are non-specific for SASP-centered inflammaging. More than that, they can be transiently modulated by acute or persistently modulated by chronic inflammatory diseases, including type 2 inflammation dermatoses in young or older populations. Meanwhile, immunosenescence and inflammaging in the skin are mutually regulated, but they do not always parallel, especially for senescent cells in the end stage. Herein, screening of reasonable indicators for inflammaging in the elderly with and/or without inflammation dermatosis *via* longitudinal data from large samples is expected.
